# Supplementary material for: In Infants with Neuroblastoma Standard Therapy Only Partially Reverts the Fecal Microbiome Dysbiosis Present at Diagnosis
Source: Microorganisms. 2025 Mar 19;13(3):691. doi: 10.3390/microorganisms13030691 (PMC11946756; doi:10.3390/microorganisms13030691)
Supplement: Supplementary file 1 [file microorganisms-13-00691-s001.zip › Supplemental Table S1_Microorganisms.pdf]

Supplemental Table S1. Differential microbial abundance in the fecal microbiomes of NB patients at onset and the ones of Healthy Controls.

NB patient samples at onset [15] *vs* Healthy Controls (HC) [17]	zero-inflated Gaussian fit		EdgeR		DESeq2		LDA	
**Taxonomy**	log2FC	FDR	log2FC	FDR	log2FC	FDR	LDA-SCORE	FDR
**Higher abundance in fecal microbiomes of NB patients at onset or lower abundance in the ones of Healthy Controls (HC).**								
p_Actinomycetota; c_Actinomycetes; o_Bifidobacteriales; **f_Bifidobacteriaceae**			3.5387	4.9E-4				
p_Actinomycetota; c_Actinomycetes; o_Bifidobacteriales; f_Bifidobacteriaceae; **g_Bifidobacterium; s_breve**	1.9716	0.0077						
p_Actinomycetota; c_Coriobacteriia; o_Eggetherellales; f_ **Eggerthellaceae**; **g_Eggerthella**	1.3651	0.0309						
p_Bacteroidota; c_Bacteroidia; o_Bacteroidales; **f_Phorphyromonadaceae**			5.8994	2.8E-4				
p_Bacteroidota; c_Bacteroidia; o_Bacteroidales; f_Tannerellaceae; **g_Parabacteroides**			3.9185	0.0162				
p_Bacteroidota; c_Bacteroidia; o_Bacteroidales; f_Tannerellaceae; **g_Parabacteroides; s_distasonis**			4.3262	0.0293				
p_Bacillota; c_Bacilli; **o_Lactobacillales**			2.4418	0.0036				
p_Bacillota; c_Bacilli; o_Lactobacillales; **f_Enterococcaceae**	8.1650	0.0023	8.165	3.6E-8	5.1819	0.0086		
p_Bacillota; c_Bacilli; o_Lactobacillales; f_Enterococcaceae**; g_Enterococcus**	5.3209	0.0025	5.3209	2.0E-4	4.9228	0.0315		
p_Bacillota; c_Bacilli; o_Lactobacillales; **f_Lactobacillaceae**			3.8121	0.0089				
p_Bacillota; c_Bacilli; o_Lactobacillales; **f_Streptococcaceae**			5.2513	8.1E-6				
p_Bacillota; c_Bacilli; o_Lactobacillales; f_Streptococcaceae; **g_Streptococcus**			2.579	0.0211				
p_Bacillota; c_Clostridia; **o_Clostridiales**			3.1396	0.0014				
p_Bacillota; c_Clostridia; o_Eubacteriales; **f_Clostridiaceae**			5.5979	5.7E-7	2.1357	0.0405		
p_Bacillota; c_Clostridia; o_Eubacteriales; f_Clostridiaceae; **g_Clostridium**			2.559	0.0162	2.2537	0.0243		
p_Bacillota; c_Clostridia; o_Eubacteriales; f_Clostridiaceae; **g_Clostridium; s_hathewayi**			5.6751	0.0014				
p_Bacillota; c_Clostridia; o_Eubacteriales; f_Clostridiaceae; **g_Clostridium; s_paraputrificum**	3.8399	0.0342	3.8399	0.0388				
p_Bacillota; c_Clostridia; o_Eubacteriales; f_ Clostridiaceae; **g_Hungatella; s_hathewayi**			4.0714	0.0095				
p_Bacillota; c_Clostridia; o_Eubacteriales; **f_Peptostreptococcaceae**			4.6151	2.8E-4				
p_Bacillota; c_Clostridia; o_Eubacteriales; f_Oscillospiraceae; **g_Anaerotruncus**			4.3168	7.5E-4				
p_Bacillota; c_Clostridia; o_Eubacteriales; f_Oscillospiraceae; **g_Anaerotruncus; s_colihominis**			4.9874	4.5E-4				
p_Bacillota; c_Negativicutes; **o_Selenomonadales**			3.0082	0.0036				
p_Bacillota; c_Negativicutes; o_Veillonellales; **f_Veillonellaceae**			4.258	4.0E-4				
p_Bacillota; c_Negativicutes; o_Veillonellales; f_Veillonellaceae; **g_Veillonella; s_dispar**	2.0306	0.0450						
p_Bacillota; c_Negativicutes; o_Veillonellales; f_Veillonellaceae; **g_Veillonella; s_tobetsuensis**	2.7349	0.0473						
**p_Pseudomonadota**	12.5145	4.2E-4	12.515	2.3E-11	1.1757	0.0384	4.58	5.1E-4
p_Pseudomonadota; **c_Deltaproteobacteria**			4.3561	0.0056				
p_Pseudomonadota; c_Deltaproteobacteria; **o_Desulfovibrionales**			4.3371	0.0036				
p_Pseudomonadota; c_Deltaproteobacteria; o_Desulfovibrionales; **f_Desulfovibrionaceae**	3.8919	0.0124	3.8919	0.0029				
p_Pseudomonadota; c_Deltaproteobacteria; o_Desulfovibrionales; f_Desulfovibrionaceae; **g_Bilophila**	4.7545	0.0034	4.7545	0.0021				
p_Pseudomonadota; c_Deltaproteobacteria; o_Desulfovibrionales; f_Desulfovibrionaceae; **g_Bilophila; s_wadsworthia**			4.004	0.0170				
p_Pseudomonadota; **c_Gammaproteobacteria**	10.7774	0.0042	10.777	5.0E-11	1.5154	0.0040	4.58	0.0037
p_Pseudomonadota; c_Gammaproteobacteria; **o_Enterobacterales**	9.8523	0.0014	9.8523	2.2E-12	1.7234	0.0054	4.59	0.0022
p_Pseudomonadota; c_Gammaproteobacteria; o_Enterobacterales; **f_Enterobacteriaceae**	8.1018	0.0012	8.1018	3.7E-11	2.3116	0.0012	4.59	0.0042
p_Pseudomonadota; c_Gammaproteobacteria; o_ Enterobacterales; f_Enterobacteriaceae; **g_Enterobacter**	7.5888	0.0056	7.5888	1.3E-5				
p_Pseudomonadota; c_Gammaproteobacteria; o_Enterobacterales; f_Enterobacteriaceae; **g_Enterobacter; s_asburiae**	6.9146	0.0016	6.9146	8.4E-5				
p_Pseudomonadota; c_Gammaproteobacteria; o_Enterobacterales; f_Enterobacteriaceae; **g_Escherichia**			4.8581	4.4E-4				
p_Pseudomonadota; c_Gammaproteobacteria; o_Enterobacterales; f_Enterobacteriaceae; **g_Escherichia; s_coli**	4.6945	0.0450	4.6945	0.0012				
p_Pseudomonadota; c_Gammaproteobacteria; o_Enterobacterales; f_Enterobacteriaceae; **g_Klebsiella**			3.5546	0.0211				
p_Pseudomonadota; c_Gammaproteobacteria; o_Enterobacterales; f_Enterobacteriaceae; **g_Klebsiella; s_variicola**			3.4136	0.0464	7.2882	0.0046		
p_Pseudomonadota; c_Gammaproteobacteria; o_Enterobacterales; f_Enterobacteriaceae; **g_Leclercia**	9.6192	8.0E-4	9.6192	6.9E-6	6.1166	0.0243		
p_Pseudomonadota; c_Gammaproteobacteria; o_Enterobacteriales; f_Enterobacteriaceae; **g_Leclercia; s_adecarboxylata**	8.001	0.0104	8.001	1.4E-4	5.9222	0.0346		

p_Pseudomonadota; c_Gammaproteobacteria; o_Enterobacteriales; f_Enterobacteriaceae; **g_Salmonella**			7.2654	8.1E-5				
p_Pseudomonadota; c_Gammaproteobacteria; o_Enterobacteriales; f_Enterobacteriaceae; **g_Salmonella; s_enterica**	6.9432	0.0137	6.9432	1.4E-4				
p_Pseudomonadota; c_Gammaproteobacteria; o_Enterobacteriales; f_Enterobacteriaceae; **g_Trabulsiella**			3.2494	0.0021				
p_Pseudomonadota; c_Gammaproteobacteria; o_Enterobacteriales; f_Enterobacteriaceae; **g_Trabulsiella; s_odontotermitis**			2.9425	0.0119				
**Higher abundance in fecal microbiome of Healthy Control (HC) or lower abundance in the ones of NB patients at onset**								
p_Actinomycetota; c_Actinomycetes; o_Bifidobacteriales; f_Bifidobacteriaceae; **g_Bifidobacterium; s_bifidum**	-1.4016	0.0138						
p_Actinomycetota; c_Actinomycetes; o_Bifidobacteriales; f_Bifidobacteriaceae; **g_Bifidobacterium; s_pseudocatenulatum**			-3.4336	0.0422				
p_Bacteroidota; c_Bacteroidia; o_Bacteroidales; f_Bacteroidaceae; **g_Bacteroides; s_ovatus**	-5.1499	2.2E-4	-5.1499	0.0012				
p_Bacteroidota; c_Bacteroidia; o_Bacteroidales; f_Bacteroidaceae; **g_Bacteroides; s_uniformis**	-2.0185	0.0054						
p_Bacteroidota; c_Bacteroidia; o_Bacteroidales; f_Bacteroidaceae; **g_ Phocaeicola; s_vulgatus**	-3.7246	0.0077						
p_Bacteroidota; c_Bacteroidia; o_Bacteroidales; **f_Prevotellaceae**	-4.1366	0.0012	-4.1366	0.0129				
p_Bacteroidota; c_Bacteroidia; o_Bacteroidales; f_Prevotellaceae; **g_Prevotella**	-5.1257	0.0029	-5.1257	0.0048	-6.9818	0.0243		
p_Bacteroidota; c_Bacteroidia; o_Bacteroidales; **f_Rikenellaceae**			-3.5311	0.0075	-5.0811	0.0402		
p_Bacillota; c_Bacilli; o_Lactobacillales; f_Lactobacillaceae; **g_Lactobacillus; s_rogosae**	-7.1605	3.5E-6	-7.1605	1.4E-4	-9.1528	0.0046		
p_Bacillota; c_Bacilli; o_Lactobacillales; f_Streptococcaceae; **g_Streptococcus; s_thermophillus**	-4.6113	0.0054	-4.6113	0.0015				
p_Bacillota; c_Clostridia; o_Eubateriales; f_Clostridiaceae; **g_Clostridium; s_aldenense**	-3.4019	0.0450	-3.4019	0.0109				
p_Bacillota; c_Clostridia; o_Eubateriales; **f_Eubacteriaceae**	-3.9262	0.0305	-3.9262	4.0E-4	-3.793	0.0221	-3.18	0.0311
p_Bacillota; c_Clostridia; o_Eubateriales; f_Eubacteriaceae; **g_Eubacterium**	-5.773	8.6E-5	-5.773	4.2E-5	-4.5786	0.0118	-3.41	0.0271
p_Bacillota; c_Clostridia; o_Eubateriales; f_Eubacteriaceae; **g_Eubacterium; s_eligens**	-8.5301	2.1E-4	-8.5301	9.5E-6	-8.0194	0.0046		
p_Bacillota; c_Clostridia; o_Eubateriales; f_Lachnospiraceae; **g_Anaerostipes**	-3.7797	0.0191	-3.7797	8.1E-4	-6.6597	0.0168		
p_Bacillota; c_Clostridia; o_Eubateriales; f_Lachnospiraceae; **g_Dorea**			-5.3419	5.8E-4				
p_Bacillota; c_Clostridia; o_Eubateriales; f_Eubacteriaceae; **g_ Eubacterium; s_hadrum**	-5.2008	2.2E-4	-5.2008	0.0012				
p_Bacillota; c_Clostridia; o_Eubateriales; f_Lachnospiraceae; **g_Roseburia**	-4.7214	0.0309	-4.7214	4.0E-4			-3.15	0.0343
p_Bacillota; c_Clostridia; o_Eubateriales; f_Lachnospiraceae; **g_Roseburia; s_faecis**	-2.6713	0.0137			-5.5196	0.0209		
p_Bacillota; c_Clostridia; o_Eubateriales; f_Lachnospiraceae; **g_Roseburia; s_intestinalis**	-2.4941	0.0080						
p_Bacillota; c_Clostridia; o_Eubateriales; f_Lachnospiraceae; **g_Roseburia; s_inulinivorans**	-4.365	0.0327	-4.365	0.0119	-7.1539	0.0080		
p_Bacillota; c_Clostridia; o_Eubateriales; f_Oscillospiraceae; **g_Faecalibacterium; s_prausnitzii**	-2.9448	0.0342						
p_Bacillota; c_Clostridia; o_Eubateriales; f_Oscillospiraceae; **g_Ruminococcus; s_bromii**	-4.7206	9.0E-4	-4.7206	0.0012	-7.5374	0.0080		
p_Bacillota; c_Clostridia; o_Eubateriales; f_Oscillospiraceae; **g_Ruminococcus; s_gauvreauii**	-1.0176	0.0328						
p_Bacillota; c_Negativicutes; o_Veillonellales; f_Veillonellaceaea; **g_Dialister**	-4.2034	0.0191	-4.2034	0.0050				
p_Bacillota; c_Negativicutes; o_Veillonellales; f_Veillonellaceaea; **g_Dialister; s_invisus**	-3.08	2.2E-4						
p_Bacillota; c_Erysipelotrichia; o_Erysipelotrichales; f_Turicibacteraceae; **g_Turicibacter**	-4.4245	0.0061	-4.4245	0.0029	-7.3208	0.0118		
p_Bacillota; c_Erysipelotrichia; o_Erysipelotrichales; f_ Turicibacteraceae; **g_Turicibacter; s_sanguinis**	-3.2298	0.0422	-3.2298	0.0320				
p_Pseudomonadota; **c_Betaproteobacteria**	-1.8199	0.0021						
p_Pseudomonadota; c_Betaproteobacteria; **o_Burkholderiales**	-1.6084	0.0380						
p_Pseudomonadota; c_Betaproteobacteria; o_Burkholderiales; **f_Sutterellaceae_**	-1.9243	0.0392			-6.7015	0.0020		
p_Pseudomonadota; c_Gammaproteobacteria; **o_Pasteurellales**					-3.5573	0.0317	-3.22	0.0265
p_Pseudomonadota; c_Gammaproteobacteria; o_Pasteurellales; **f_Pasteurellaceae**							-3.22	0.0338
p_Pseudomonadota; c_Gammaproteobacteria; o_Pasteurellales; f_Pasteurellaceae; **g_Haemophilus**	-1.6309	0.0472						
p_Pseudomonadota; c_Gammaproteobacteria; o_Pasteurellales; f_Pasteurellaceae; **g_Haemophilus; s_parainfluenzae**	-1.1812	0.0450						
p_Pseudomonadota a; c_Gammaproteobacteria; o_Pasteurellales; f_Pasteurellaceae; **g_Haemophilus; s_sputorum**	-3.3546	0.0042	-3.3546	0.0412	-7.9367	0.0046		
p_Pseudomonadota; c_Gammaproteobacteria; o_Pasteurellales; f_Pasteurellaceae; **g_Mannheimia**	-1.7943	0.0449						
p_Pseudomonadota; c_Gammaproteobacteria; o_Pasteurellales; f_Pasteurellaceae; **g_Mannheimia; s_varigena**	-1.4604	0.0342						

The number in square brackets indicates the number of patients in each group. The columns represent the statistical analyses using four algorithms (the zero-inflated Gaussian Fit, the EdgeR, the DESeq2, and the LDA). All statistical analyses showed the FDR (False Discovery Rate) that indicates the p-value after adjustment for multiple comparisons. FDR equal to or less than 0.05 was considered statistically significant. All statistical analyses adjust the data for

imbalanced class distribution (under-sampling) and dataset sparsity. The taxonomy is shown as p_Phylum; c_Class; o_Order; f_Family; g_Genus; s_Specie. The base two logarithmic value of fold changes (log2FC) represents the increase (+) or decrease (-) in the abundance of a particular taxon between the two groups. The LDA-Score represents the effect size of each abundant taxa.
