# Supplementary material for: In Infants with Neuroblastoma Standard Therapy Only Partially Reverts the Fecal Microbiome Dysbiosis Present at Diagnosis
Source: Microorganisms. 2025 Mar 19;13(3):691. doi: 10.3390/microorganisms13030691 (PMC11946756; doi:10.3390/microorganisms13030691)
Supplement: Supplementary file 1 [file microorganisms-13-00691-s001.zip › Supplemental Table S2_Microorganisms.pdf]

Supplemental Table S2. Differential abundance in the fecal microbiomes of NB patients after 2 cycles of therapy and of healthy children's samples.

NB patients after 2 cycles of therapy [5] *vs.* Healthy Children [17]	zero-inflated Gaussian fit		EdgeR		DESeq2		LDA	
**Taxonomy**	log2FC	FDR	log2FC	FDR	log2FC	FDR	LDA-SCORE	FDR
**Higher abundance in the microbiomes of NB patients after 2 cycles of therapy or lower in the ones of healthy children's (HC) samples**								
*p_Bacteroidota; c_Bacteroidia; o_Bacteroidales; **f_Porphyromonadaceae***			4.0291	0.0311				
*p_Bacillota; **c_Bacilli***			3.2279	0.0011	2.0624	0.0230		
*p_Bacillota; c_Bacilli; **o_Lactobacillales***			3.4161	0.0015				
*p_Bacillota; c_Bacilli; o_Lactobacillales; **f_Carnobacteriaceae***			3.471	0.0201				
*p_Bacillota; c_Bacilli; o_Lactobacillales; f_Carnobacteriaceae; **g_Granulicatella***			2.9934	0.0370				
*p_Bacillota; c_Bacilli; o_Lactobacillales; f_Carnobacteriaceae; **g_Granulicatella; s_adiacens***			3.2611	0.0234				
*p_Bacillota; c_Bacilli; o_Lactobacillales; **f_Enterococcaceae***			8.0837	7.1E-9	6.3788	0.0122		
*p_Bacillota; c_Bacilli; o_Lactobacillales; f_Enterococcaceae; **g_Enterococcus***	6.2389	0.0031	6.2389	1.8E-5				
*p_Bacillota; c_Bacilli; o_Lactobacillales; **f_Streptococcaceae***			4.5695	7.5E-5	3.3194	0.0038		
*p_Bacillota; c_Bacilli; o_Lactobacillales; f_Streptococcaceae; **g_Streptococcus***			2.69	0.0370	2.9056	0.0368		
*p_Bacillota; c_Bacilli; o_Lactobacillales; f_Streptococcaceae; **g_Streptococcus; s_pseudopneumoniae***	6.0868	0.0183	6.0868	2.6E-5				
*p_Bacillota; c_Clostridia; **o_Eubacteriales***			2.2627	0.0351				
*p_Bacillota; c_Clostridia; o_Eubacteriales; **f_Clostridiaceae***			2.451	0.0414				
*p_Bacillota; c_Clostridia; o_Eubacteriales; f_Clostridiaceae; **g_Clostridium***			2.6604	0.0427				
*p_Bacillota; c_Clostridia; o_Eubacteriales; f_Lachnospiraceae; **g_Enterocloster; s_bolteae***			4.1756	0.0225				
*p_Bacillota; c_Clostridia; o_Eubacteriales; f_Clostridiaceae; **g_Clostridium; s_clostridioforme***	5.2257	0.0023	5.2257	0.0091				
*p_Bacillota; c_Clostridia; o_Eubacteriales; f_Oscillospiraceae; **g_Ruminococcus***			5.171	1.3E-4				
*p_Bacillota; c_Clostridia; o_Eubacteriales; f_Lachnospiraceae; **g_Mediterraneibacter; s_faecis***			8.5563	2.0E-4				
*p_Bacillota; c_Negativicutes; o_Veillonellales; f_Veillonellaceae**; g_Veillonella; s_dispar***	3.712	0.0024	3.712	0.0225				
*p_Fusobacteriota; c_Fusobacteriia; o_Fusobacteriales; f_Fusobacteriaceae; **g_Fusobacterium***	3.023	0.0442						
p_Pseudomonadota			4.2733	4.5E-10	1.5639	0.0339		
*p_Pseudomonadota; c_Epsilonproteobacteria; o_Campylobacterales; f_Campylobacteraceae; **g_Campylobacter***	7.9988	0.0114	7.9988	2.9E-6				
*p_Pseudomonadota; c_Epsilonproteobacteria; o_Campylobacterales; f_Campylobacteraceae; **g_Campylobacter; s_concisus***			7.1855	2.6E-5	7.5975	0.0319		
*p_Pseudomonadota; **c_Gammaproteobacteria***			4.6539	7.4E-9	2.847	2.9E-4		
*p_Pseudomonadota; c_Gammaproteobacteria; **o_Enterobacterales***			5.4181	1.8E-10	3.073	4.5E-4		
*p_Pseudomonadota; c_Gammaproteobacteria; o_Enterobacterales; **f_Enterobacteriaceae***			5.1186	8.0E-6	3.4442	0.0014		
*p_Pseudomonadota; c_Gammaproteobacteria; o_Enterobacterales; f_Enterobacteriaceae; **g_Cronobacter***			2.9217	0.0499				
*p_Pseudomonadota; c_Gammaproteobacteria; o_Enterobacterales; f_Enterobacteriaceae; **g_Escherichia***			6.3227	6.7E-5				
*p_Pseudomonadota; c_Gammaproteobacteria; o_Enterobacterales; f_Enterobacteriaceae; **g_Escherichia; s_coli***			6.1417	8.5E-6				
*p_Pseudomonadota; c_Gammaproteobacteria; o_Enterobacterales; f_Enterobacteriaceae; **g_Salmonella***			3.0573	0.0370				
*p_Pseudomonadota; c_Gammaproteobacteria; o_Enterobacterales; f_Enterobacteriaceae; **g_Yokenella***			5.8085	6.7E-5				
*p_Pseudomonadota; c_Gammaproteobacteria; o_Enterobacterales; f_Enterobacteriaceae; **g_Yokenella; s_regensburgei***			4.6623	0.0055				
**Higher abundance in the microbiomes of healthy childrens sample or lower in the ones of NB patients after 2 cycles of therapy**								
*p_Actinomycetota; c_Actinomycetes; o_Bifidobacteriales; f_Bifidobacteriaceae; **g_Bifidobacterium; s_adolescentis***	-8.3346	1.7E-4	-8.3346	0.0234	-25.213	4.5E-15		
*p_Actinomycetota; c_Actinomycetes; o_Bifidobacteriales; f_Bifidobacteriaceae; **g_Bifidobacterium; s_bifidum***	-8.2136	0.0028	-8.2136	0.0153	-6.6396	0.0148		
*p_Actinomycetota; c_Actinomycetes; o_Bifidobacteriales; f_Bifidobacteriaceae; **g_Bifidobacterium; s_dentium***	-10.753	0.0186	-10.753	0.0225	-25.703	7.1E-17		
*p_Actinomycetota; c_Actinomycetes; o_Bifidobacteriales; f_Bifidobacteriaceae; **g_Bifidobacterium; s_pseudocatenulatum***			-7.5856	0.0380				
*p_Actinomycetota; c_Coriobacteriia; **o_Coriobacteriales***					-5.6737	0.0429		
*p_Bacteroidota; c_Bacteroidia; o_Bacteroidales; f_Bacteroidaceae; **g_Bacteroides; s_caccae***	-7.0316	0.0024						
*p_Bacteroidota; c_Bacteroidia; o_Bacteroidales; f_Bacteroidaceae; **g_Bacteroides; s_fragilis***	-11.911	3.2E-4	-11.911	0.0225	-8.1959	0.0319		
*p_Bacteroidota; c_Bacteroidia; o_Bacteroidales; f_Bacteroidaceae; **g_Bacteroides; s_thetaiotaomicron***	-9.2783	6.1E-5	-9.2783	0.0234				
*p_Bacteroidota; c_Bacteroidia; o_Bacteroidales; f_Bacteroidaceae; **g_Bacteroides; s_uniformis***	-5.4271	0.0110						

p_Bacteroidota; c_Bacteroidia; o_Bacteroidales; f_Bacteroidaceae; **g_Phocaeicola; s_vulgatus**	-6.5943	0.0167						
p_Bacteroidota; c_Bacteroidia; o_Bacteroidales; **f_Prevotellaceae**	-5.0879	0.0227						
p_Bacteroidota; c_Bacteroidia; o_Bacteroidales; f_Prevotellaceae; **g_Prevotella**	-5.8694	0.0262						
p_Bacteroidota; c_Bacteroidia; o_Bacteroidales; f_Prevotellaceae; **g_Prevotella; s_copri**	-4.6768	0.0057						
p_Bacteroidota; c_Bacteroidia; o_Bacteroidales; f_Rikenellaceae; **g_Alistipes; s_onderdonkii**	-4.1386	0.0057						
p_Bacillota; c_Clostridia; o_Eubacteriales; f_Oscillospiraceae; **g_Gemminger**	-8.6101	0.0122	-8.6101	0.0370				
p_Bacillota; c_Clostridia; o_Eubacteriales; f_Oscillospiraceae; **g_Gemminger; s_formicilis**	-7.5247	6.7E-4						
p_Bacillota; c_Bacilli; o_Lactobacillales; f_Lactobacillaceae; **g_Lactobacillus**			-6.7438	0.0370				
p_Bacillota; c_Bacilli; o_Lactobacillales; f_Lactobacillaceae; **g_Lactobacillus; s_rogosae**	-6.0258	0.0031						
p_Bacillota; c_Clostridia; o_Eubacteriales; f_Lachnospiraceae; **g_Enterocloster; s_aldenense**	-6.799	1.8E-4	-6.799	0.0234	-6.6112	0.0426		
p_Bacillota; c_Clostridia; o_Eubacteriales; f_Lachnospiraceae; **g_ Enterocloster; s_asparagiforme**	-4.3181	0.0016			-6.942	0.0470		
p_Bacillota; c_Clostridia; o_Eubacteriales; f_Lachnospiraceae; **g_Blautia; s_luti**	-3.8204	0.0068						
p_Bacillota; c_Clostridia; o_Eubacteriales; f_Lachnospiraceae; **g_Blautia; s_producta**	-6.0837	1.8E-4	-6.0837	0.0386	-25.534	5.3E-15		
p_Bacillota; c_Clostridia; o_Eubacteriales; f_Lachnospiraceae; **g_Blautia; s_wexlerae**	-7.7167	0.0024	-7.7167	0.0094	-10.372	1.4E-6		
p_Bacillota; c_Clostridia; o_Eubacteriales; f_Lachnospiraceae; **g_Dorea**	-8.0657	0.0057	-8.0657	0.0370	-22.777	7.2E-12		
p_Bacillota; c_Clostridia; o_Eubacteriales; f_Eubacteriaceae; **g_Anaerostipes; s_hadrus**	-7.2344	0.0057	-7.2344	0.0225	-5.5014	0.0319		
p_Bacillota; c_Clostridia; o_Eubacteriales; f_Clostridiaceae; **g_Hungathella; s_hathewayi**	-4.8315	0.0380			-7.6095	0.0461		
p_Bacillota; c_Clostridia; o_Eubacteriales; f_Lachnospiraceae; **g_Roseburia; s_inulinivorans**	-8.5416	1.8E-4	-8.5416	0.0272	-23.007	4.6E-14		
p_Bacillota; c_Clostridia; o_Clostridiales; **f_Oscillospiraceae**	-5.398	0.0141			-22.277	5.1E-12		
p_Bacillota; c_Clostridia; o_Eubacteriales; f_Oscillospiraceae; **g_Anaerotruncus; s_colihominis**	-2.7117	0.0025						
p_Bacillota; c_Clostridia; o_Eubacteriales; f_Oscillospiraceae; **g_Ruminococcus**			-4.5914	0.0370	-3.8949	0.0212		
p_Bacillota; c_Clostridia; o_Eubacteriales; f_Oscillospiraceae; **g_Ruminococcus; s_bromii**	-6.007	1.8E-4	-6.007	0.0386				
p_Bacillota; c_Clostridia; o_Eubacteriales; f_Oscillospiraceae; **g_Ruminococcus; s_torques**	-6.9024	0.0081	-6.9024	0.0234	-24.24	3.4E-18		
p_Bacillota; **c_Erysipelotrichia**					-3.0371	0.0011		
p_Bacillota; c_Erysipelotrichia; **o_Erysipelotrichales**			-3.1956	0.0433	-3.3707	4.5E-4		
p_Bacillota; c_Erysipelotrichia; o_Erysipelotrichales; **f_ Erysipelotrichaceae**					-2.8034	0.0058		
p_Bacillota; c_Erysipelotrichia; o_Erysipelotrichales; f_Coprobabillaceae; **g_Thomasclavelia; s_cocleata**	-5.9689	0.0165	-5.9689	0.0153				
p_Bacillota; c_Erysipelotrichia; o_Erysipelotrichales; f_Coprobabillaceae; **g_Thomasclavelia; s_ramosum**	-5.8116	0.0102	-5.8116	0.0153	-4.304	0.0461		
p_Bacillota; c_Erysipelotrichia; o_Eubacteriales; f_Eubacteriaceae; **g_Eubacterium**	-5.1452	0.0442	-5.1452	0.0499	-8.3191	0.0212		
p_Bacillota; c_Erysipelotrichia; o_Eubacteriales; f_Eubacteriaceae; **g_Eubacterium; s_dolichum**	-4.7515	0.0233			-7.7531	0.0319		
p_Bacillota; c_Negativicutes; o_Veillonellales; f_Veillonellaceae; **g_Dialister**	-8.4214	6.1E-4	-8.4214	0.0281	-8.3741	0.0315		
p_Bacillota; c_Negativicutes; o_Veillonellales; f_Veillonellaceae; **g_Dialister; s_invisus**	-7.888	6.1E-5	-7.888	0.0234	-8.0973	0.0398		
p_Pseudomonadota; c_Betaproteobacteria; o_Burkholderiales; f_Sutterellaceae; **g_Sutterella; s_wadsworthensis**	-4.4562	0.0081						
p_Pseudomonadota; c_Gammaproteobacteria; o_Enterobacterales; f_Enterobacteriaceae; **g_Citrobacter; s_werkmanii**	-4.4562	0.0129						
p_Pseudomonadota; c_Gammaproteobacteria; o_Enterobacterales; f_Enterobacteriaceae; **g_Leclercia; s_adecarboxylata**	-0.6396	0.0167						
p_Verrucomicrobiota; c_Verrucomicrobiia; o_Verrucomicrobiales; f_Akkermansiaceae; **g_Akkermansia**			-7.8577	0.0370	-22.088	1.8E-10		

The number in square brackets indicates the number of patients in each group. The columns represent the statistical analyses using four algorithms (the zero-inflated Gaussian Fit, the EdgeR, the DESeq2, and the LDA). All statistical analyses showed the FDR (False Discovery Rate) that indicates the p-value after adjustment for multiple comparisons. FDR equal to or less than 0.05 was considered statistically significant. The taxonomy is shown as p_Phylum; c_Class; o_Order; f_Family; g_Genus; s_Specie. The base two logarithmic value of fold changes (log2FC) represents the increase (+) or decrease (-) in the abundance of a particular taxon between the two groups. The LDA-Score represents the effect size of each abundant taxa.
