# Supplementary material for: In Infants with Neuroblastoma Standard Therapy Only Partially Reverts the Fecal Microbiome Dysbiosis Present at Diagnosis
Source: Microorganisms. 2025 Mar 19;13(3):691. doi: 10.3390/microorganisms13030691 (PMC11946756; doi:10.3390/microorganisms13030691)
Supplement: Supplementary file 1 [file microorganisms-13-00691-s001.zip › Supplemental Table S3_Microorganisms.pdf]

Supplemental Table S3. Differential microbial abundance in the fecal microbiomes of NB patients after 4 cycles of therapy or the ones from healthy children.

**NB patients after 4 cycles of therapy [6] *vs* HC-Controls [17]**	**zero-inflated Gaussian fit**		**EdgeR**		**DESeq2**		**LDA**	
**Taxonomy**	log2FC	FDR	log2FC	FDR	log2FC	FDR	LDA-SCORE	FDR
**Higher abundance in the fecal microbiomes of NB patients at the end of therapy or lower abundance in the ones of healthy children's samples (HC-Controls)**								
p_Bacteroidota; c_Bacteroidia; o_Bacteroidales; f_Bacteroidaceae; **g_Bacteroides; s_xylanisolvens**			6.1531	0.0021				
**p_Bacillota**	4.1877	0.0496	4.1877	1.4E-8				
p_Bacillota; **c_Bacilli**			12.492	1.8E-15				
p_Bacillota; c_Bacilli; **o_Lactobacillales**			9.9159	3.3E-11				
p_Bacillota; c_Bacilli; o_Lactobacillales; **f_Enterococcaceae**			17.503	1.2E-14				
p_Bacillota; c_Bacilli; o_Lactobacillales; f_Enterococcaceae; **g_Enterococcus**			13.524	2.4E-11				
p_Bacillota; c_Bacilli; o_Lactobacillales; **f_Streptococcaceae**			9.0566	2.2E-9	4.3344	0.0100		
p_Bacillota; c_Bacilli; o_Lactobacillales; f_Streptococcaceae; **g_Streptococcus**			6.8355	6.4E-7				
p_Bacillota; c_Bacilli; o_Lactobacillales; f_Streptococcaceae; **g_Streptococcus; s_thermophilus**			8.3448	3.3E.4				
p_Bacillota; c_Clostridia; o_Eubacteriales; f_Clostridiaceae; **g_Clostridium; s_paraputrificum**			5.2546	0.0233				
p_Bacillota; c_Clostridia; o_Eubacteriales; f_ Lachnospiraceae; **g_ Enterocloster**			3.7105	0.0386				
p_Bacillota; c_Clostridia; o_Eubacteriales; f_ Lachnospiraceae; **g_** Enterocloster **; s_clostridioformis**			4.5914	0.0084				
p_Bacillota; c_Erysipelotrichia; o_Erysipelotrichales; f_Coprobabillaceae; **g_Thomasclavelia; s_spiroformis**			4.4034	0.0050				
p_Pseudomonadota; **c_Deltaproteobacteria**	2.2086	11.0535						
p_Pseudomonadota; c_Deltaproteobacteria; o_Desulfovibrionales; f_Desulfovibrionaceae; **g_Bilophila; s_wadsworthia**			5.5002	0.0260				
p_Pseudomonadota; c_Gammaproteobacteria; o_Enterobacterales; f_Enterobacteriaceae; **g_Kosakonia**			5.5432	0.0014				
p_Pseudomonadota; c_Gammaproteobacteria; o_Enterobacterales; f_Enterobacteriaceae; **g_Kosakonia; s_sacchari**			6.0017	0.0021				
**Higher abundance in the fecal microbiomes healthy children's samples (HC-Controls) or lower abundance in the ones of NB patients at the end of therapy**								
**p_Actinomycetota**			-3.0847	0.0389				
p_Bacillota; c_Clostridia; o_Eubacteriales; f_Lachnospiraceae; **g_Roseburia; s_inulinivorans**			-7.8959	0.0332				
p_Bacillota; c_Clostridia; o_Eubacteriales; f_Lachnospiraceae; **g_Dorea**	-7.3729	0.0121			-30.0	2.7E-21		
p_Bacillota; c_Erysipelotrichia; o_Erysipelotrichales; f_Coprobacillaceae; **g_Thomasclavelia; s_ramosa**			-4.6923	0.0332				
p_Bacillota; c_Erysipelotrichia; o_Erysipelotrichales; f_Turicibacteraceae; **g_Turicibacter**	-7.1817	0.0121						
p_Psedomonadota; **c_Alphaproteobacteria**	-6.5252	13.0631			-27.331	1.3E-15		
p_Pseudomonadota; c_Alphaproteobacteria; **o_** Hyphomicrobiales	-7.6444	0.0011			-26.277	3.7E-15		
p_Pseudomonadota; c_Alphaproteobacteria; o_ Hyphomicrobiales; **f_Hyphomicrobiaceae**	-7.0950	0.0023			-30.0	1.0E-21		
p_Pseudomonadota; c_Gammaproteobacteria; o_Pasteurellales; f_ Pasteurellaceae; **g_Haemophilus; s_sputorum**			-7.3858	0.0332				
p_Verrucomicrobiota; c_Verrucomicrobiia; o_Verrucomicrobiales; f_Akkermansiaceae; **g_Akkermansia**	-6.5777	0.0121			-29.84	2.2E-22		

The number in square brackets indicates the number of patients in each group. The columns represent the statistical analyses using four algorithms (the zero-inflated Gaussian Fit, the EdgeR, the DESeq2, and the LDA). All statistical analyses showed the FDR (False Discovery Rate) that indicates the p-value after adjustment for multiple comparisons. FDR equal to or less than 0.05 was considered statistically significant. The taxonomy is shown as p_Phylum; c_Class;

o_Order; f_Family; g_Genus; s_Specie. The base two logarithmic value of fold changes (log2FC) represents the increase (+) or decrease (-) in the abundance of a particular taxon between the two groups. The LDA-Score represents the effect size of each abundant taxa.
