# Supplementary material for: In Infants with Neuroblastoma Standard Therapy Only Partially Reverts the Fecal Microbiome Dysbiosis Present at Diagnosis
Source: Microorganisms. 2025 Mar 19;13(3):691. doi: 10.3390/microorganisms13030691 (PMC11946756; doi:10.3390/microorganisms13030691)
Supplement: Supplementary file 1 [file microorganisms-13-00691-s001.zip › Supplemental Table S4_Microorganisms.pdf]

Supplemental Table S4. Differential abundance in the fecal microbiomes of NB patients after 4 cycles of therapy and of NB patients at onset.

NB patients at the end of therapy [6] *vs.* NB patients at onset [15]	zero-inflated Gaussian fit		EdgeR		DESeq2		LDA	
**Taxonomy**	log2FC	FDR	log2FC	FDR	log2FC	FDR	LDA-SCORE	FDR
**Higher abundance in the fecal microbiomes of NB patients at end of therapy or lower in the ones of NB patients at the onset**								
p_Actinomycetota; c_Actinomycetes; ***o_Actinomycetales***			6.4431	0.0016				
p_Actinomycetota; c_Actinomycetes; o_Micrococcales; ***f_Micrococcaceae***			4.7143	0.0296				
p_Bacteroidota; c_Bacteroidia; o_Bacteroidales; f_Bacteroidaceae; ***g_Bacteroides; s_ovatus***	4.6411	0.0170	4.6411	0.0041	9.186	0.0332		
p_Bacillota			5.2692	1.5E-8				
p_Bacillota; ***c_Bacilli***			11.829	3.1E-10				
p_Bacillota; c_Bacilli; ***o_Lactobacillales***			9.3106	1.2E-8	5.0486	7.1E-4		
p_Bacillota; c_Bacilli; o_Lactobacillales; ***f_Enterococcaceae***			9.7544	9.4E-5				
p_Bacillota; c_Bacilli; o_Lactobacillales; f_Enterococcaceae; ***g_Enterococcus***			9.0494	2.5E-4				
p_Bacillota; c_Bacilli; o_Lactobacillales; ***f_Streptococcaceae***			5.5173	0.0104	5.9089	0.0119		
p_Bacillota; c_Bacilli; o_Lactobacillales; f_Streptococcaceae; ***g_Streptococcus***			5.3874	0.0165				
p_Bacillota; c_Bacilli; o_Lactobacillales; f_Streptococcaceae; ***g_Streptococcus; s_thermophilus***	12.638	0.0143	12.638	7.4E-8	13.366	9.9E-4		
p_Bacillota; c_Clostridia; o_Eubacteriales; ***f_Eubacteriaceae***			6.8077	9.4E-5				
p_Bacillota; c_Clostridia; o_Eubacteriales; f_Eubacteriaceae; ***g_Eubacterium***			5.6969	0.0012				
p_Bacillota; c_Clostridia; o_Eubacteriales; f_Lachnospiraceae; ***g_Lachnospira; s_eligens***	7.3422	0.0170	7.3422	6.5E-4	9.9892	0.0215		
p_Bacillota; c_Clostridia; o_Eubacteriales; f_Lachnospiraceae; ***g_Blautia; s_wexlerae***			4.2355	0.0330				
p_Bacillota; c_Clostridia; o_Eubacteriales; ***f_Oscillospiraceae***			3.7018	0.0280				
p_Bacillota; c_Clostridia; o_Eubacteriales; f_Oscillospiraceae; ***g_Faecalibacterium; s_prausnitzii***	0.5812	0.0482						
p_Bacillota; c_Clostridia; o_Eubacteriales; f_Oscillospiraceae; ***g_Gemminger***	-5.377	0.0035						
p_Bacillota; c_Clostridia; o_Eubacteriales; f_Oscillospiraceae; ***g_Gemminger; s_formicilis***	-8.9181	0.0137	-8.9181	0.0330				
p_Pseudomonadota; ***c_Betaproteobacteria***	1.6073	0.0286						
**Higher abundance in NB samples at onset or lower in the ones of NB patients at end of therapy**								
p_Actinomycetota; c_Actinomycetes; o_Bifidobacteriales; f_Bifidobacteriaceae; ***g_Bifidobacterium; s_breve***	-7.2717	0.0078	-7.2717	0.0330	-8.6188	0.0215		
p_Actinomycetota; c_Actinomycetes; o_Bifidobacteriales; f_Bifidobacteriaceae; ***g_Bifidobacterium; s_longum***	-8.5872	0.0087	-8.5872	0.0273				
p_Bacillota; c_Clostridia; o_Eubacteriales; f_Peptostreptococcaceae; ***g_Clostridioides; s_difficile***			-5.5242	0.0444				
p_Bacillota; c_Clostridia; o_Eubacteriales; f_Clostridiaceae; ***g_Hungatella; s_hathewayi***			-11.006	0.0273				
p_Bacillota; c_Clostridia; o_Eubacteriales; f_Clostridiaceae; ***g_Clostridium; s_neonatale***	-12.448	2.9E-4	-12.448	0.0151	-28.765	1.8E-16		
p_Bacillota; c_Erysipelotrichia; o_Erysipelotrichales; f_Coprobacillaceae; ***g_Thomasclavelia s_ramosum***	-3.4354	0.0482						
p_Bacillota; c_Negativicutes; o_Veillonellales; Veillonellaceae; ***g_Veillonella; s_atypica***	-7.3498	0.0482						
p_Bacillota; c_Negativicutes; o_Veillonellales; Veillonellaceae; ***g_Veillonella; s_parvula***			-7.5665	0.0330				
p_Pseudomonadota			-8.577	0.0158				
p_Pseudomonadota; c_Gammaproteobacteria; o_Enterobacterales; f_Enterobacteriaceae; ***g_Pseudescherichia; s_vulneris***	-1.9753	0.0482						
p_Pseudomonadota; c_Gammaproteobacteria; o_Enterobacterales; f_Enterobacteriaceae; ***g_Leclercia***	-12.909	0.0394	-12.909	0.0217	-29.304	2.2E-17		

The number in square brackets indicates the number of patients in each group. The columns represent the statistical analyses using four algorithms (the zero-inflated Gaussian Fit, the EdgeR, the DESeq2, and the LDA). All statistical analyses showed the FDR (False Discovery Rate) that indicates the p-value after adjustment for multiple comparisons. FDR equal to or less than 0.05 was considered statistically significant. The taxonomy is shown as p_Phylum; c_Class; o_Order; f_Family; g_Genus; s_Specie. The base two logarithmic value of fold changes (log2FC) represents the increase (+) or decrease (-) in the abundance of a particular taxon between the two groups. The LDA-Score represents the effect size of each abundant taxa.
