# Supplementary material for: In Infants with Neuroblastoma Standard Therapy Only Partially Reverts the Fecal Microbiome Dysbiosis Present at Diagnosis
Source: Microorganisms. 2025 Mar 19;13(3):691. doi: 10.3390/microorganisms13030691 (PMC11946756; doi:10.3390/microorganisms13030691)
Supplement: Supplementary file 1 [file microorganisms-13-00691-s001.zip › Supplemental Table S5_Microorganisms.pdf]

## Supplemental Table S5. Differential abundance in the fecal microbiomes of NB patients after 4 and 2 cycles of therapy.

| NB patients after 4 cycles of therapy [5] *vs.* NB patients after 2 cycles of drugs treatment [6] | zero-inflated Gaussian fit | | EdgeR | | DESeq2 | | LDA | |
|---|---|---|---|---|---|---|---|---|
| Taxonomy | log2FC | FDR | log2FC | FDR | log2FC | FDR | LDA-SCORE | FDR |
| **Higher abundance in the fecal microbiomes of NB patients after 4 cycles of therapy or lower in the ones of NB patients after 2 cycles of therapy** | | | | | | | | |
| p_Actinomycetota; c_Actinomycetes; o_Bifidobacteriales; f_Bifidobacteriaceae; **g_Bifidobacterium; s_bifidum** | | | 6.4199 | 0.0380 | | | | |
| p_Actinomycetota; c_Actinomycetes; o_Bifidobacteriales; f_Bifidobacteriaceae; **g_Bifidobacterium; s_pseudocatenulatum** | | | 6.3698 | 0.0380 | | | | |
| | | | | | | | | |
| p_Bacteroidota; c_Bacteroidia; o_Bacteroidales; f_Bacteroidaceae; **g_Bacteroides; s_uniformis** | | | 8.5738 | 0.0380 | | | | |
| | | | | | | | | |
| p_Bacillota; c_Clostridia; o_Eubacteriales; f_Clostridiaceae; **g_Clostridium; s_butyricum** | | | 9.529 | 0.0380 | | | | |
| p_Bacillota; c_Clostridia; o_Eubacteriales; f_Clostridiaceae; **g_Clostridium; s_innocuum** | | | 6.3895 | 0.0380 | | | | |
| p_Bacillota; c_Clostridia; o_Eubacteriales; f_Clostridiaceae; **g_Clostridium; s_paraputrificum** | | | 8.9336 | 0.0398 | | | | |
| p_Bacillota; c_Clostridia; o_Eubacteriales; f_Lachnospiraceae; **g_Anaerostipes; s_hadrus** | | | 6.4286 | 0.0499 | | | | |
| p_Bacillota; c_Clostridia; o_Eubacteriales; f_Lachnospiraceae; **g_Blautia; s_producta** | | | 5.7424 | 0.0380 | | | | |
| p_Bacillota; c_Clostridia; o_Eubacteriales; f_Lachnospiraceae; **g_Blautia; s_wexlerae** | | | 10.678 | 0.0339 | 26.305 | 1.5E-16 | | |
| p_Bacillota; c_Clostridia; o_Eubacteriales; f_Lachnospiraceae; **g_Roseburia; s_faecis** | | | 7.7252 | 0.0339 | | | | |
| p_Bacillota; c_Clostridia; o_Eubacteriales; f_Oscillospiraceae; **g_Ruminococcus; s_bromii** | | | 10.09 | 0.0339 | | | | |
| p_Bacillota; c_Clostridia; o_Eubacteriales; f_Oscillospiraceae; **g_Ruminococcus; s_torques** | 8.0091 | 0.0031 | 8.0091 | 0.0339 | 24.066 | 3.6E-14 | | |
| p_Bacillota; c_Clostridia; o_Eubacteriales; f_Oscillospiraceae; **g_Flavonifractor; s_plautii** | | | 7.5719 | 0.0380 | | | | |
| p_Bacillota; c_Negativicutes; o_Acidaminococcales; **f_Acidaminococcaceae** | | | 8.3712 | 0.0315 | 25.839 | 1.6E-16 | | |
| | | | | | | | | |
| p_Pseudomonadota; c_Deltaproteobacteria; o_Desulfovibrionales; f_Desulfovibrionaceae; **g_Bilophila; s_wadsworthia** | | | 7.7555 | 0.0380 | | | | |
| p_Pseudomonadota; c_Gammaproteobacteria; o_Enterobacterales; f_Enterobacteriaceae; **g_Kosakonia; s_sacchari** | | | 7.6877 | 0.0380 | | | | |
| **Higher abundance in the fecal microbiomes of NB patients after 2 cycles of therapy or lower in the ones of NB patients after 4 cycles of therapy** | | | | | | | | |
| p_Actinomycetota; c_Actinomycetes; o_Micrococcales; **f_Micrococcaceae** | | | | | -8.9935 | 0.0132 | | |
| p_Actinomycetota; c_Actinomycetes; o_Micrococcales s; f_Micrococcaceae; **g_Rothia** | | | | | -8.4708 | 0.0403 | | |
| | | | | | | | | |
| p_Bacteroidota; c_Bacteroidia; o_Bacteroidales; **f_Prevotellaceae** | -3.6094 | 0.0042 | | | -28.799 | 1.7E-20 | | |
| p_Bacteroidota; c_Bacteroidia; o_Bacteroidales; f_Prevotellaceae; **g_Prevotella** | | | -27.418 | 3.2E-18 | -28.725 | 5.3E-20 | | |
| | | | | | | | | |
| p_Bacillota; c_Clostridia; o_Eubacteriales; f_Clostridiaceae; **g_Clostridium; s_disporicum** | | | -5.8843 | 0.0380 | | | | |
| p_Bacillota; c_Clostridia; o_Eubacteriales; f_Lachnospiraceae; **g_Roseburia; s_intestinalis** | -6.3531 | 0.0055 | | | | | | |
| p_Bacillota; c_Erysipelotrichia; o_Erysipelotrichales; f_Turicibacteraceae; **g_Turicibacter; s_sanguinis** | | | | | -25.412 | 7.3E-16 | | |
| p_Bacillota; c_Negativicutes; o_Veillonellales; f_Veillonellaceaea; **g_Veillonella; s_atypica** | | | -5.6708 | 0.0380 | -8.7678 | 0.0049 | | |
| | | | | | | | | |
| | | | | | | | | |
| p_Pseudomonadota; **c_Epsilonproteobacteria** | -7.7298 | 5.7E-5 | -7.7298 | 0.0304 | -26.281 | 3.4E-17 | | |
| p_Pseudomonadota; c_Epsilonproteobacteria; **o_Campylobacterales** | -8.8736 | 4.6E-4 | -8.8736 | 0.0375 | -28.171 | 1.4E-19 | | |
| p_Pseudomonadota; c_Epsilonproteobacteria; o_Campylobacterales; **f_Campylobacteraceae** | -7.6404 | 0.0042 | | | -30.0 | 6.7E-22 | | |
| p_Pseudomonadota; c_Epsilonproteobacteria; o_Campylobacterales; f_Campylobacteraceae; **g_Campylobacter** | | | | | -27.162 | 3.5E-18 | | |
| p_Pseudomonadota; c_Epsilonproteobacteria; o_Campylobacterales; f_Campylobacteraceae; **g_Campylobacter; s_concisus** | | | -9.4906 | 0.0297 | -26.772 | 4.1E-17 | | |

The number in square brackets indicates the number of patients in each group. The columns represent the statistical analyses using four algorithms (the zero-inflated Gaussian Fit, the EdgeR, the DESeq2, and the LDA). All statistical analyses showed the FDR (False Discovery Rate) that indicates the p-value after adjustment for multiple comparisons. FDR equal to or less than 0.05 was considered statistically significant. The taxonomy is shown as p_Phylum; c_Class; o_Order; f_Family; g_Genus; s_Specie. The base two logarithmic value of fold changes (log2FC) represents the increase (+) or decrease (-) in the abundance of a particular taxon between the two groups. The LDA-Score represents the effect size of each abundant taxa.
