# Supplementary material for: In Infants with Neuroblastoma Standard Therapy Only Partially Reverts the Fecal Microbiome Dysbiosis Present at Diagnosis
Source: Microorganisms. 2025 Mar 19;13(3):691. doi: 10.3390/microorganisms13030691 (PMC11946756; doi:10.3390/microorganisms13030691)
Supplement: Supplementary file 1 [file microorganisms-13-00691-s001.zip › Supplemental Table S6_Microorganisms.pdf]

Supplemental Table S6. Supervised analysis with Random Forest algorithm.

A. NB patients at the Onset [15] *vs* Healthy children (HC) [17]

Taxa	Phylum	Mean Decrease Accuracy	OBB error	Abundance Higher in	Specificity	Sensitivity
Enterobacteriaceae	P	0.0672	0.219	NB onset	0.79	0.78
Roseburia	F	0.0309	0.156	HC	0.86	0.83
Eubacterium	F	0.0298	0.156	HC	0.86	0.83
Eubacteriaceae	F	0.0247	0.219	HC	0.79	0.78
Enterocloster clostridioformis	F	0.0221	0.250	HC	0.77	0.74
Anaerostipes hadrus	F	0.0188	0.250	HC	0.77	0.74
Blautia	F	0.0186	0.156	HC	0.86	0.83
Erysipelotrichaceae	F	0.0173	0.219	NB onset	0.79	0.78
Pasteurellaceae	P	0.0151	0.219	HC	0.79	0.78
Enterococcaceae	F	0,0129	0.219	NB onset	0.79	0.78
Oscillospiraceae	F	0.0116	0.219	HC	0.79	0.78
Desulfovibrionaceae	P	0.0116	0.219	NB onset	0.79	0.78
Porphyromonadaceae	B	0.0116	0.219	NB onset	0.79	0.78
Ruminococcus	F	0.0111	0.156	HC	0.86	0.83
Enterococcus	F	0.0100	0.156	NB onset	0.86	0.83

B. NB patients after 2 cycles of Therapy [5] *vs* Healthy children (HC) [17]

Taxa	Phylum	Mean Decrease Accuracy	OBB error	Abundance Higher in	Specificity	Sensitivity
Enterococcaceae	F	0.0334	0.182	2 cycles therapy	0.81	1.00
Enterococcus	F	0.0280	0.227	2 cycles therapy	0.80	0.50
Erysipelotrichaceae	F	0.0179	0.182	HC	0.81	1.00
Enterobacteriaceae	P	0.0168	0.273	2 cycles therapy	0.81	1.00
Peptostreptococcaceae	F	0.0157	0.182	2 cycles therapy	0.81	1.00

C. NB patients after 4 cycles of Therapy [6] *vs* Healthy children (HC) [17]

Taxa	Phylum	Mean Decrease Accuracy	OBB error	Abundance Higher in	Specificity	Sensitivity
Bifidobacterium	A	0.0187	0.174	HC	0.81	1.00
Veillonella	F	0.0184	0.174	4 cycles Therapy	0.81	1.00
Bifidobacteriaceae	A	0.0133	0.174	HC	0.81	1.00
Bacteroidaceae	B	0.0131	0.174	HC	0.81	1.00

Random Forest classifications are presented in order of importance, based on the Mean Decrease Accuracy values. Patients' microbiomes (at onset and after two or four cycles of therapy) were consistently compared to those of age-matched healthy children. Various comparisons identify different taxa associated with patients or healthy controls. The classifications indicate the phylum to which each taxon belongs, along with the corresponding Out-of-Bag (OOB) error, specificity, and sensitivity values. The microorganisms are classified based on the phylum to which they belong: *Actinomycetota* (A), *Bacteroidota* (B), *Bacillota* (F), *Pseudomonadota* (P).
