# Supplementary figures and images for: In Infants with Neuroblastoma Standard Therapy Only Partially Reverts the Fecal Microbiome Dysbiosis Present at Diagnosis
Source: Microorganisms. 2025 Mar 19;13(3):691. doi: 10.3390/microorganisms13030691 (PMC11946756; doi:10.3390/microorganisms13030691)

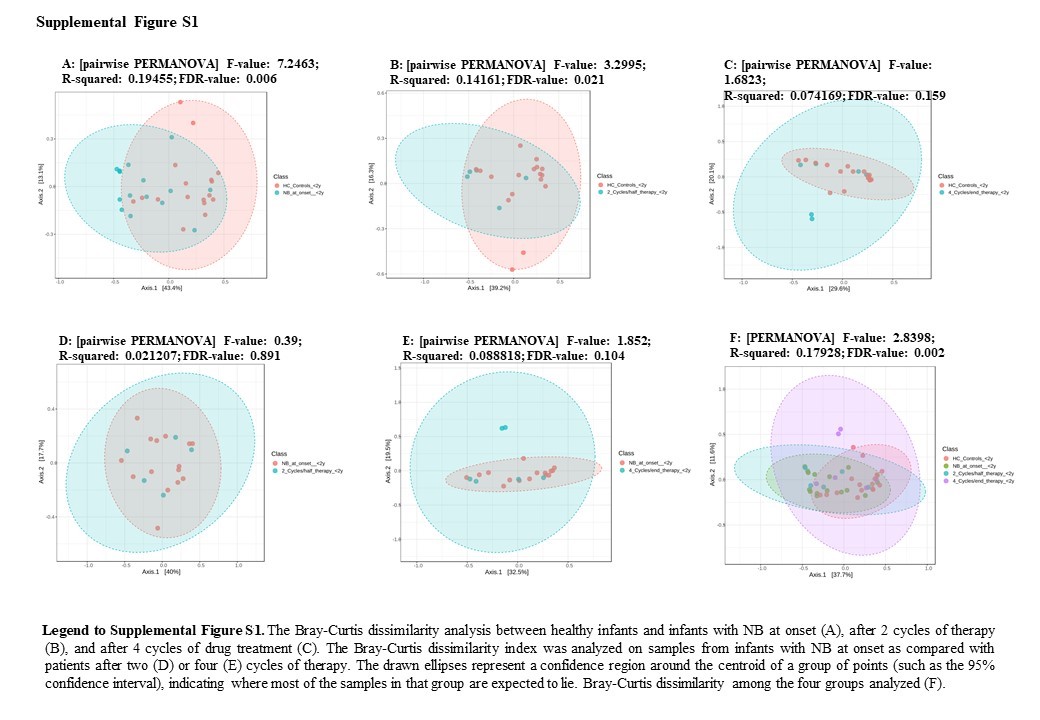

Supplement: Supplementary file 1 [file microorganisms-13-00691-s001.zip › Supplemental Figure S1_Microorganisms.jpg]

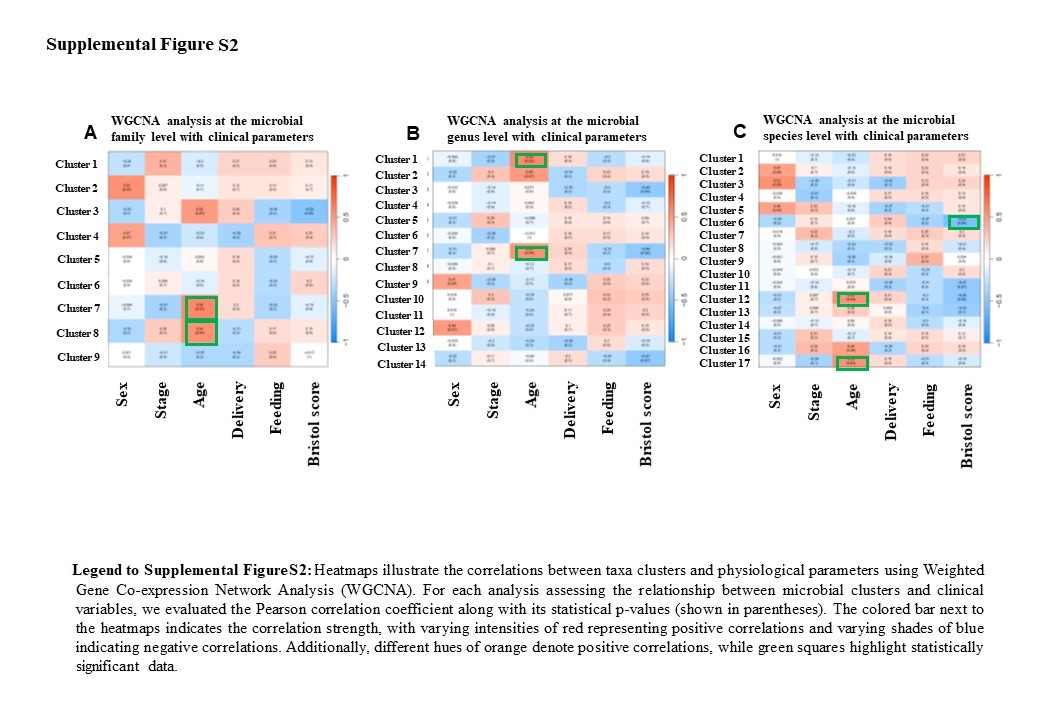

Supplement: Supplementary file 1 [file microorganisms-13-00691-s001.zip › Supplemental Figure S2_Microorganisms.jpg]
